# Surface Chemistry-Driven Surface-Enhanced Raman Scattering Fingerprinting of Aptamers on Silver Colloids

**DOI:** 10.1021/acsaom.6c00151

**Published:** 2026-07-09

**Authors:** Sara Pandolfi, Elisabetta Venuti, Erica Locatelli, Xavier Mateos, Nicolas Pazos-Perez, Tommaso Salzillo, Luca Guerrini

**Affiliations:** † Department of Industrial Chemistry “Toso Montanari”, 9296 University of Bologna, via Piero Gobetti 85, 40129 Bologna, Italy; ‡ Physics and Crystallography of Materials Research Group, FiCMA, 16777 University Rovira i Virgili, Marcel·lí Domingo 1, 43007 Tarragona, Spain; § Department of Physical and Inorganic Chemistry, University Rovira i Virgili, Marcel·lí Domingo 1, 43007 Tarragona, Spain

**Keywords:** surface-enhanced Raman scattering (SERS), aptamers, nanoparticle surface chemistry, silver colloids, adsorption regime, ratiometric analysis

## Abstract

The surface-enhanced Raman scattering (SERS) spectra of nucleic acids (NAs) often vary substantially across plasmonic substrates. However, systematic comparative studies across the most commonly employed plasmonic platforms remain limited, particularly for structurally complex NAs, such as aptamers. Herein, we investigate the direct SERS fingerprinting of a thiolated aptamer across three representative silver colloidal systems widely used in nucleic-acid SERS studies: intrinsically cationic spermine-modified nanoparticles, post-synthetically spermine-modified citrate colloids, and chloride-modified citrate colloids. The results show that, under the investigated conditions, the surface chemistry appears to primarily determine the dominant adsorption regime, modulating the balance between the backbone electrostatics and nucleobase–metal interactions. Intrinsically cationic nanoparticles preserve folding-dependent spectral differences, whereas negatively charged systems exhibit pronounced coverage-dependent spectral variability, highlighting the sensitivity of the interfacial SERS response to surface coverage and preparation history. These findings highlight that the SERS fingerprint of the aptamer in colloidal suspension largely emerges from the interplay between the structural complexity of the NA and the adsorption regime imposed by the nanoparticle surface chemistry. Therefore, different colloidal substrates can provide complementary spectroscopic information.

## Graphical abstract

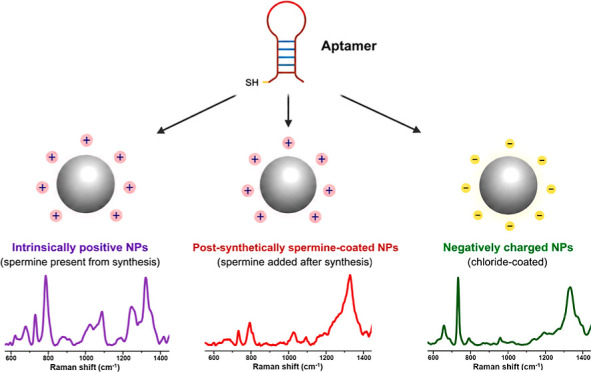

## Introduction

The detection and characterization of nucleic acids (NAs), including DNA, RNA, and functional oligonucleotides such as aptamers, are essential in modern analytical chemistry and molecular biology as well as in biomedical diagnostics. Identifying, quantifying, and monitoring these biomolecules constitute essential preliminary steps for many applications ranging from pathogen detection to precision medicine. However, traditional techniques, such as the polymerase chain reaction and fluorescence, often present experimental difficulties that can limit their field applicability.^1^

Over the past few decades, plasmonic nanomaterials have emerged as key enabling platforms for chemical sensing and biomedical analysis, owing to their ability to confine and amplify electromagnetic fields at the nanoscale.^2,3^ In this context, surface-enhanced Raman scattering (SERS) has become one of the most powerful plasmon-assisted analytical techniques, providing molecularly specific vibrational fingerprints with high sensitivity and minimal sample preparation.^4,5^ Unlike many conventional analytical methods, SERS is inherently compatible with complex biological samples and offers the potential for rapid, label-free analysis, while its response is strongly governed by nanoscale surface chemistry and interfacial interactions.^6,7^

SERS-based detection strategies can be broadly classified into indirect and direct approaches, depending on whether the analytical signal originates from extrinsic Raman reporters or from the vibrational fingerprints of the target analyte itself.^8^ Indirect SERS methods have generally been demonstrated to be more readily adaptable to quantitative and multiplexed detection schemes, particularly when operating in complex biological environments. In contrast, direct SERS interrogation enables access to chemically rich and structurally informative vibrational signatures but faces intrinsic limitations that become particularly pronounced in real matrixes. In such environments, competing species and nonspecific adsorption can hide or distort the spectral contribution of the target analyte, ultimately restricting the applicability of direct SERS as a robust and generalizable sensing strategy. These challenges are further exacerbated in the case of biomolecules, such as NAs, whose structural complexity and conformational variability often result in variable or poorly reproducible SERS responses.^9^

Recent advances in artificial intelligence have begun to directly address several intrinsic limitations associated with the direct SERS interrogation of biomolecules. Machine learning and deep learning approaches enable the analysis of high-dimensional and highly convoluted vibrational data sets, allowing subtle spectral patterns and correlations to be extracted even when individual spectral features are weak, overlapped, or affected by variability in substrate morphology and measurement conditions.^10^ In the context of SERS, AI-assisted strategies have demonstrated the ability to classify biological samples, discriminate closely related molecular signatures, and perform quantitative analyses directly from label-free spectra acquired in complex biological environments.^11,12^ This data-driven paradigm opens new opportunities for deploying direct SERS as a viable biosensing strategy. Importantly, the effectiveness and generalizability of AI-assisted SERS critically depend on a rigorous understanding of how the molecular structure, surface chemistry, and plasmonic interactions collectively shape the recorded spectral response. This highlights the need to better understand the physicochemical surface–biomolecule interactions.

Direct SERS of NAs has been extensively reported in the literature, with most studies relying on colloidal silver nanoparticles as plasmonic substrates for measurements performed in suspension.^9,13^ Broadly, these colloidal systems can be classified according to their surface chemistry and charge. Positively charged substrates, most notably spermine-coated silver nanoparticles (AgSp), have been widely employed to promote electrostatic adhesion with the negatively charged phosphate backbone of NA, thereby facilitating biomolecular adsorption and NA-induced aggregation, leading to the formation of plasmonic hotspots that enhance the SERS response.^9,14–16^ A large body of work has also focused on the combination of aggregation-inducing agents and negatively charged colloids modified with halide anions (e.g., chloride, bromide, or iodide).^13,17–19^ Halide-assisted surface modification has been shown to further improve spectral quality by suppressing background signals and modulating DNA–surface interactions.^19^ On the other hand, multivalent cations such as Mg^2+^, Ca^2+^, or Al^3+^ present in the aggregating agents play a dual role: they partially screen the electrostatic repulsion between NA and the negatively charged metal surface while simultaneously promoting colloidal aggregation of the colloids.^9^ Collectively, these studies highlight the strong dependence of the SERS response on surface chemistry, charge mediation, and aggregation conditions. Notably, a recent work by Deriu and Fabris, employing a dinucleotide model on gold nanostars post-synthetically modified with spermine, has further highlighted the specificity and complexity of DNA building block adsorption at the nanoparticle interface.^20^

In direct SERS interrogation of NAs, aptamers have received comparatively limited attention, despite their widespread use in biosensing, diagnostics, and targeted delivery.^21,22^ NA aptamers are short, synthetic DNA or RNA oligonucleotides identified through in vitro selection procedures, most commonly based on the systematic evolution of ligands by exponential enrichment (SELEX).^21,22^ Aptamers fold into well-defined secondary and tertiary structures stabilized by intramolecular base pairing and noncovalent interactions.^21,22^ Binding events are typically accompanied by conformational rearrangements of the aptamer scaffold.^21,22^ In this context, aptamers represent a nontrivial case for direct SERS interrogation, as their conformational landscape involves dynamic folding equilibria and environment-responsive structural rearrangements, giving rise to multiple possible adsorption regimes at the nanoparticle interface.

Intrinsic SERS spectra of these synthetic oligonucleotides have been mostly reported in the context of SERS-based detection of a broad range of target analytes (e.g., small molecules, toxins, proteins, and viral components), where thiolated aptamers were covalently anchored to negatively charged gold or silver plasmonic surfaces via formation of the covalent metal–sulfur bond.^23–28^ In these studies, detection is typically based on SERS spectral changes associated with the formation of the aptamer–analyte complex, arising from a combination of conformational rearrangements of the aptamer and the emergence of new vibrational contributions linked to the proximity of the bound analyte to the metal surface.^23,26–29^ Owing to their well-defined secondary and tertiary structures, aptamers are expected to exhibit interaction modes with plasmonic surfaces that are highly sensitive to the surface chemistry of the underlying colloids, with direct implications for their SERS response. On this basis, we systematically investigate the direct SERS fingerprinting of an aptamer in suspension using plasmonic silver colloids with distinct surface chemistry. Specifically, positively charged systems were examined by comparing intrinsically cationic nanoparticles obtained through spermine incorporation during synthesis (AgSp) with citrate-stabilized colloids rendered cationic via post-synthetic spermine adsorption (AgCitSp), while chloride-modified citrate colloids (AgCitCl) were employed as a well-defined negatively charged control. To this end, a model aptamer system was selected by taking inspiration from a previously reported RNA aptamer successfully exploited for the early detection of bladder cancer lesions in a preclinical model.^30^ By comparatively analyzing the spectral response of unfolded and hairpin conformations across these platforms, we aim to elucidate how nanoparticle surface properties influence the interfacial configuration and shapes the resulting interfacial vibrational signatures. Through combined qualitative and ratiometric analysis, we show that, in the investigated systems, the direct SERS fingerprint of the aptamer largely emerges from the interplay between the structural complexity of the NA and the adsorption regime imposed by the nanoparticle surface chemistry. In this framework, interfacial electrostatics, surface functionality, and coverage-dependent reorganization processes collectively define the vibrational response of the surface-bound complex.

## Experimental Section

### Materials

l-Ascorbic acid (AA, ≥99%), silver nitrate (AgNO_3_, ≥99%), spermine tetrahydrochloride (SpCl_4_, 99%), sodium borohydride (NaBH_4_, >96%), phosphate-buffered saline (PBS, tablets, ≥99%), branched polyethylenimine (PEI, average molecular weight ∼25,000, ≥99%), sodium citrate (98%), magnesium sulfate (MgSO_4_, ≥99%), potassium chloride (KCl, 99%), and aluminum nitrate (Al­(NO_3_)_3_, ≥99%) were obtained from Merck and used as received. TCEP hydrochloride (TCEP, >99.97%) was purchased from MedChemExpress. The 34-nucleotide DNA aptamer was purchased from Eurofins Genomics (Germany). Ultrapure water (Milli-Q, 18.2 MΩ cm) was used to prepare all aqueous solutions, and all glassware was cleaned with aqua regia prior to the experiments.

### Preparation of Aptamer Samples

The aptamer was initially dissolved in Milli-Q water to a concentration of 100 μM. From this stock solution, two equimolar working solutions (5 μM, 100 μL) were prepared to obtain the aptamer in two distinct conformational ensembles with different degrees of folding: a less folded (extended-enriched) state and a folded (hairpin-enriched) state. For both conformations, the working solutions were subsequently subdivided into 20 μL aliquots to avoid repeated freeze–thaw cycles prior to use. For the preparation of the less folded aptamer conformation (uApt), an aliquot of the aptamer solution in Milli-Q water was heated to 95 °C for 10 min and then rapidly cooled by immersion in an ice bath (thermal shock), a treatment that favors an extended, unfolded configuration. To prepare the hairpin-enriched aptamer (hApt), a second aliquot of the same aptamer solution was prepared in PBS (pH 7.4). The solution was heated to 95 °C for 10 min and subsequently allowed to cool passively to room temperature (25 °C) in approximately 2 h, thereby promoting equilibration toward thermodynamically favored folded conformations dominated by intramolecular hairpin structures. After thermal treatment, both uApt and hApt working solutions were divided into small single-use aliquots and stored at −20 °C. For each experiment, only the required aliquot was thawed at room temperature immediately before use, and thawed aliquots were not refrozen. To assess whether the freeze–thaw storage step induced detectable changes in the aptamer ensemble, UV–vis spectra were recorded immediately after thermal treatment and after frozen storage at −20 °C followed by thawing at room temperature. No appreciable changes in the absorption profile were observed, supporting the absence of major freeze–thaw-induced changes detectable by UV–Vis spectroscopy under the present conditions. For control experiments involving thiol reduction, hApt solutions (5 μM in PBS) were incubated with TCEP using a 10:1 molar excess relative to the aptamer for 60 min at 35 °C prior to incubation with the colloidal substrates.

### Synthesis of Positively Charged Silver Colloids (AgSp)

Before use, glass vials were functionalized with PEI by immersion in an aqueous PEI solution (0.2% v/v) for approximately 12 h, followed by extensive rinsing with Milli-Q water to remove the loosely bound polymer.^31^ In 10 mL of Milli-Q water, 20 μL of a 0.5 M silver nitrate (AgNO_3_) solution was added under stirring, followed by the addition of 7 μL of a 0.1 M spermine tetrahydrochloride solution (SpCl_4_). After stirring the mixture for 20 min at room temperature, 250 μL of a freshly prepared 0.01 M sodium borohydride (NaBH_4_) solution was rapidly injected to initiate the reduction of silver ions. The colloidal suspensions were kept undisturbed overnight at room temperature and protected from light. During this period, a small amount of sediment was observed at the bottom of the vial, which was attributed to a fraction of nanoparticles forming aggregates. Prior to use, the supernatant containing the colloidal dispersion was carefully collected by pipetting and separated from the sediment. The synthesis yielded a nanoparticle concentration of 0.3 nM, calculated using the maximum absorbance at 393 nm and ε = 1.7 × 10^10^ M^–1^ cm^–1^, as summarized in Table S1. All AgSp colloids were systematically employed within 24 h after synthesis, and any remaining material was discarded.

### Synthesis of Negatively Charged Citrate-Reduced Silver Colloids (AgCit)

Citrate-reduced silver colloids (AgCit) were synthesized by modifying a previously published protocol.^32^ Briefly, AA (100 μL, 0.1 M) and sodium citrate (1364 μL, 0.1 M) solutions were added under vigorous stirring to 100 mL of Milli-Q water maintained at its boiling temperature. After 1 min, a mixed solution containing AgNO_3_ and magnesium sulfate (MgSO_4_), prepared by combining 298 μL of a 0.1 M AgNO_3_ solution and 224 μL of a 0.1 M MgSO_4_ solution, was added to the reaction mixture. The resulting suspension was kept under reflux for an additional 60 min and then allowed to cool to room temperature. Excess citrate was removed by centrifugation (5400 rpm, 30 min). Unless otherwise stated, the resulting nanoparticle pellet was redispersed in an equal volume of Milli-Q water (pH ∼ 6.5). As for AgSp colloids, AgCit colloids were also employed the day after preparation.

### Surface Modification of AgCit Silver Colloids: Spermine-Induced Charge Reversal of AgCit Colloids (AgCitSp)

Surface charge reversal of citrate-reduced silver colloids was carried out by adapting a previously reported polyamine-based charge reversal protocol.^33^ After centrifugation, performed as described above, and removal of the supernatant, the AgCit nanoparticle pellet was redispersed in half the initial volume of Milli-Q water prior to ligand exchange, yielding a nanoparticle concentration of approximately 0.08 nM, calculated using the maximum absorbance at 446 nm and ε = 7.1 × 10^10^ M^–1^ cm^–1^, as summarized in Table S1. Subsequently, in a PEI-pretreated glass vial, 20 μL of a 0.1 M spermine tetrahydrochloride solution was added to 3 mL of Milli-Q water. Under vigorous stirring, 2 mL of the concentrated AgCit suspension was added to the spermine solution. After complete addition, the suspension was stirred for 10 min at room temperature, yielding positively charged spermine-modified silver colloids (AgCitSp) dispersed in a medium at a pH of approximately 6.1, with a final concentration of 0.03 nM, calculated using the maximum absorbance at 446 nm and ε = 7.1 × 10^10^ M^–1^ cm^–1^, as noted in Table S1.

### Surface Modification of AgCit Silver Colloids: Chloride-Coated Colloids (AgCitCl)

For halide surface modification, AgCit colloids were redispersed in the original volume, with a concentration of 0.05 nM, calculated using the maximum absorbance at 446 nm and ε = 7.1 × 10^10^ M^–1^ cm^–1^, as summarized in Table S1. Subsequently, they were incubated overnight with potassium chloride by mixing 1900 μL of AgCit with 100 μL of a 12 mM KCl solution, following a previously reported procedure,^34^ to yield a final nanoparticle concentration of approximately 0.04 nM, calculated using the maximum absorbance at 446 nm and ε = 7.1 × 10^10^ M^–1^ cm^–1^, as summarized in Table S1.

### Sample Preparation for Surface-Enhanced Raman Scattering

For SERS measurements, 120 μL of the colloidal silver suspensions were mixed with appropriate volumes (from 6 to 13.3 μL) of uApt or hApt solutions (5 μM or 0.5 μM), resulting in final aptamer concentrations ranging from 25 nM to 500 nM, with an associated dilution of the nanoparticle concentration of approximately 5–10% across the explored conditions. Although DNA-induced AgSp clusters may slowly sediment upon standing, they remain weakly bound and can be readily redispersed before measurement, restoring a homogeneous suspension, as previously reported for related AgSp assemblies.^15^ Prior to SERS acquisition, the samples were gently redispersed by brief vortexing, followed by momentary immersion in a 42 kHz ultrasonic bath for approximately 1 s. This step was used solely to homogenize the colloidal suspension immediately before measurement. For negatively charged colloids, aggregation of the preincubated colloid–aptamer mixtures was triggered immediately prior to measurement by adding 10 μL of a 0.5 M MgSO_4_ solution or, as an alternative aggregating agent, 10 μL of a 0.5 M Al­(NO_3_)_3_ solution. SERS spectra were acquired shortly thereafter. All measurements were performed in two independent experimental series, each including duplicate acquisitions.

### Instrumentation

Secondary-structure predictions were performed using the OligoAnalyzer tool (Integrated DNA Technologies, IDT), based on nearest-neighbor thermodynamic parameters, using ionic conditions approximating PBS buffer (pH 7.4). Optical and morphological characterizations of the nanomaterials were performed using UV–vis spectroscopy with an Agilent Varian Cary 5000 spectrophotometer and transmission electron microscopy (TEM) with a JEOL JEM-1011 microscope operating at 100 kV. TEM samples were prepared by drop-casting the colloidal suspensions onto carbon–Formvar-coated 200-mesh copper grids and allowing them to dry under ambient conditions. ζ-Potential measurements were carried out using a Malvern Zetasizer Nano instrument. SERS experiments were performed on colloidal suspensions using a Renishaw inVia Raman system equipped with 514, 633, and 785 nm excitation lasers, with powers at the objective of 3 mW, 4 mW, and 30 mW, respectively. A 2400 lines/mm diffraction grating was used for measurements at 514 nm, whereas a 1200 lines/mm grating was employed for 633 and 785 nm excitations. The laser beam was focused on the bulk of the colloidal suspension using a macrolens collector with a working distance of 15 mm. For all the excitation wavelengths, each spectrum was acquired with an integration time of 10 s and 5 accumulations. Data processing and analysis were performed using ImageJ, Microsoft Excel, and Origin software. All SERS spectra were baseline-corrected prior to analysis. For visualization purposes, spectra were subsequently smoothed using a Savitzky–Golay filter (20-point window, third-order polynomial) implemented in Origin 2024. Quantitative analyses, including ratiometric intensity calculations, were performed on baseline-corrected spectra prior to smoothing.

## Results and Discussion

### Model Aptamer System: Conformational States and Structural Features

A model aptamer system was selected by taking inspiration from a previously reported urine-stable 34-mer thiol-terminated 2′-fluoro-pyrimidine RNA aptamer targeting integrin α5β1, which has been successfully conjugated to gold nanorods for the early detection of bladder cancer lesions in a preclinical model.^30^ The DNA analogue of this aptamer was selected as a structurally related model system derived from the previously reported RNA sequence, in which modified nucleobases were replaced with canonical DNA bases, thereby enabling a more accessible and reproducible platform for the present study.

Figure 1 shows the sequence of the 34-mer aptamer, which was functionalized at the 5′ end with a C6-thiol linker sequence. Two distinct conformational ensembles with different degrees of folding were investigated: a less folded (extended-enriched) state and a hairpin-enriched state. In the first case, an aliquot of the aptamer dissolved in Milli-Q water was subjected to a thermal-shock protocol, thereby kinetically favoring an extended conformation (uApt) characterized by reduced intramolecular base pairing.^35^ In contrast, to promote intramolecular base pairing with folding into a hairpin conformation (hApt), a second aliquot of the aptamer was prepared in PBS and subjected to thermal treatment followed by slow cooling to room temperature, which promotes equilibration toward thermodynamically favored folded conformations (Figure S1).

**Figure 1:**
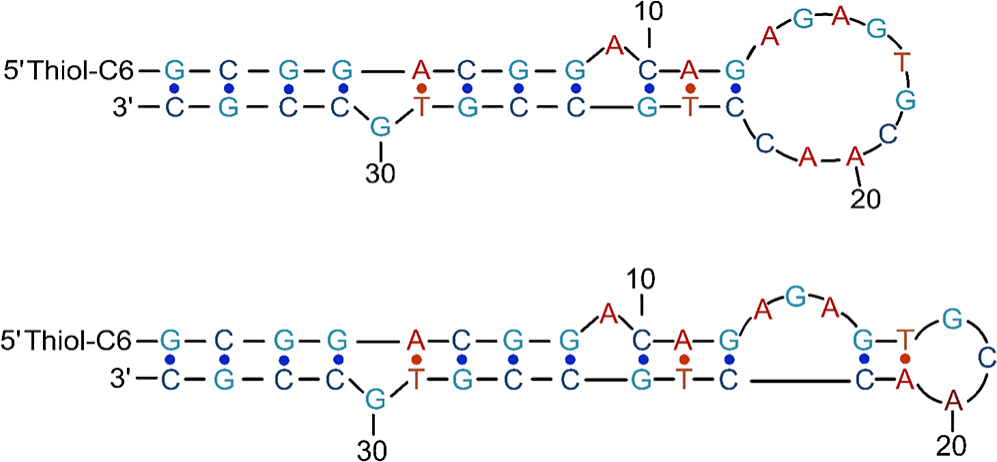
Predicted secondary structures of the thiolated aptamer 5′-HS-(CH_2_)_6_-GCGGACGGACAGAGAGTGCAACCTGCCGTGCCGC-3′. The two lowest-energy hairpin conformations (structures 1, top; and 2, bottom) obtained from secondary structure prediction are shown. Under the selected conditions (25 °C, PBS pH 7.4), the estimated free energies were Δ*G* = −7.3 and −6.2 kcal/mol for structures 1 and 2, respectively. These values should be interpreted qualitatively, as they depend on the assumed ionic conditions and model parameters.

Secondary-structure predictions indicate two low-energy hairpin conformations (structures 1 and 2), with structure 1 being thermodynamically favored (Δ*G* = −7.3 kcal/mol), compared to structure 2 (Δ*G* = −6.2 kcal/mol). Importantly, the apical loop is enriched in adenine residues, whereas the stem region is predominantly composed of guanine–cytosine base pairs, which is consistent with a stable stem–loop architecture and a structurally flexible loop domain. Under our experimental conditions (25 °C, PBS pH 7.4), these data suggest that hairpin 1 is expected to be the predominant fold in solution, whereas hairpin 2 may contribute as a minor subpopulation. Predicted populations should be interpreted qualitatively, as they depend on the specific ionic environment and limitations of the folding model. On the other hand, intermolecular homoduplex formation is expected to be negligible under the investigated conditions, where unimolecular folding is favored over bimolecular hybridization, as widely reported for DNA systems in which the intramolecular secondary structure competes with duplex formation.^36^

Based on simple geometric estimates, uApt has a contour length of approximately ∼20 nm in the fully extended limit, while its effective end-to-end dimension in solution is expected to be substantially shorter due to chain flexibility, with an effective diameter on the order of ∼1–1.5 nm.^37,38^ In contrast, hApt adopts a more compact structure with a characteristic axial length of ∼5–7 nm and a diameter of approximately ∼2 nm, consistent with a short duplex stem.^39^ We anticipate that, upon interaction with the colloidal surface, the aptamer forms a surface-bound complex that intrinsically perturbs its solution-state conformation, to an extent that primarily depends on the surface chemistry of the colloid.

### Silver Colloids and Their Surface Chemistry

Three classes of plasmonic silver colloidal systems with distinct surface chemistries were selected for this study as they represent well-established yet fundamentally different approaches to control nanoparticle surface charge and size, both of which are critical parameters in SERS-based NA analysis. The first system (AgSp) consists of intrinsically cationic silver nanoparticles synthesized in the presence of spermine tetrahydrochloride (SpCl_4_) and using borohydride as a reducing agent in the presence of SpCl_4_, yielding positively charged nanoparticles.^31^ The second system comprises citrate-stabilized silver nanoparticles (AgCit) rendered cationic via post-synthetic adsorption of SpCl_4_. As a negatively charged control, AgCit nanoparticles modified with chloride ions were chosen because chloride anions are present at the surface of both cationic colloids, thereby isolating the role of the surface charge from that of the halide.

The conventional AgSp synthetic approach produces small nanoparticles with an average particle diameter of 30 ± 10 nm, characterized by an extinction maximum centered at approximately 393 nm (Figures 2 and S2), and a strongly positive ζ-potential (+39 mV).^31^ AgSp colloids are known to provide intense SERS signals of NA when excited with green laser lines, whereas extremely weak or nondetectable signals are typically observed when NIR excitation wavelengths are employed.^31^ This behavior is attributed to the limited ability of these small nanoparticles to sustain plasmonic modes efficiently coupled to longer wavelengths, resulting in weak electromagnetic field enhancement even upon aggregation.^41^ Importantly, in AgSp colloids, spermine molecules are present during nanoparticle nucleation and growth, acting simultaneously as capping agents and growth modulators. Consequently, spermine is restrained at the metal surface, contributing to the definition of the surface chemistry from the earliest stages of particle formation.

**Figure 2:**
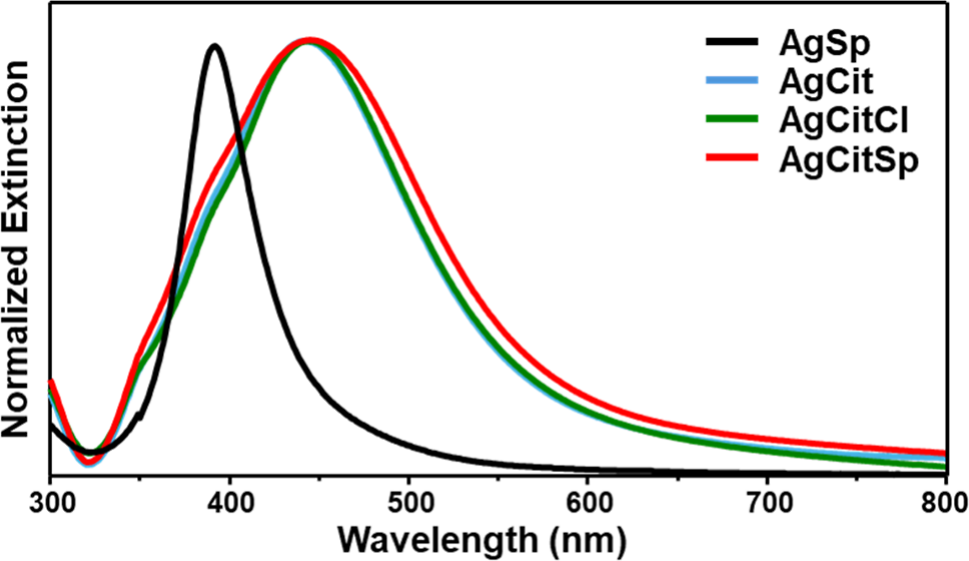
Normalized extinction spectra of the colloidal systems (AgSp, AgCit, AgCitSp, and AgCitCl).

The synthesis of colloidally stable Ag silver nanoparticles that are intrinsically capped with short polyamine molecules, but with significantly larger sizes, remains extremely challenging. Therefore, we adopted a complementary strategy based on post-synthetic surface charge manipulation of large, negatively charged silver nanoparticles.^33^ First, citrate-capped silver colloids (AgCit) were synthesized, yielding nanoparticles with an average diameter of ∼70 ± 10 nm (Figure S2), a negative ζ-potential of approximately −40 mV, and a localized surface plasmon resonance centered at 446 nm (Figure 2). Subsequently, surface charge reversal was achieved by post-synthetic adsorption of spermine molecules onto AgCit colloids, yielding spermine-modified nanoparticles (AgCitSp). The amount of SpCl_4_ added to AgCit colloids was carefully optimized to introduce the minimum quantity required to achieve surface charge reversal and stable ζ-potential (+25 mV) as well as retain colloidal stability while deliberately avoiding excess free spermine molecules in the bulk solution. Under these conditions, the nominal amount of SpCl_4_ added to the bulk, normalized to the total available metallic surface area, was estimated to be approximately 6-fold higher than that used for AgSp colloids. Similarly, halide modification of AgCit was performed by adding a controlled amount of KCl, sufficient to suppress residual citrate-related bands in the SERS spectra while remaining low enough to prevent significant nanoparticle aggregation, as described in a previously reported procedure.^15^

The background SERS spectra of the investigated colloids are presented in Figure 3. To yield intense signals, the aggregation of colloidal suspensions was induced using a magnesium sulfate solution. MgSO_4_ acts as a passive aggregating agent, promoting nanoparticle coupling through electrostatic screening without specifically interacting with the silver surface or altering its surface chemistry.^41^ In the ∼400–1800 cm^–1^ spectral range (Figure 3A), the SERS spectrum of AgCit aggregated with MgSO_4_ exhibits intense bands characteristic of surface-bound citrate and residual nitrate species.^42^ These contributions disappear in AgCitCl and AgCitSp colloids, consistent with surface displacement by chloride ions, which are well-known to compete strongly for adsorption sites on silver surfaces.^43^ This interpretation is further corroborated by the analysis of the low-wavenumber region (Figure 3B). For AgCit, we observe a broad band centered at ∼226 cm^–1^, which is attributed to the convolution of multiple surface Ag–O vibrational contributions, arising from oxygen-coordinated species such as citrate and ascorbate derivatives bound to the silver surface, as well as from minor oxide-like Ag–O domains generated during nanoparticle synthesis.^44^ Upon addition of chloride (AgCitCl), a distinct and intense band emerges at ∼238 cm^–1^, which is assigned to the ν­(Ag–Cl) stretching mode.^42,43^ Notably, in the presence of spermine (both AgSp and AgCitSp colloids), this band shifted from ∼238 to ∼243 cm^–1^. Similar results were observed when AgCit was aggregated by adding sufficient amounts of KCl and SpCl_4_. This displacement highlights a modification of the local coordination environment of the surface-bound chloride, consistent with the electrostatic interactions between the protonated amine groups of spermine and Cl^–^ at the silver interface.

**Figure 3:**
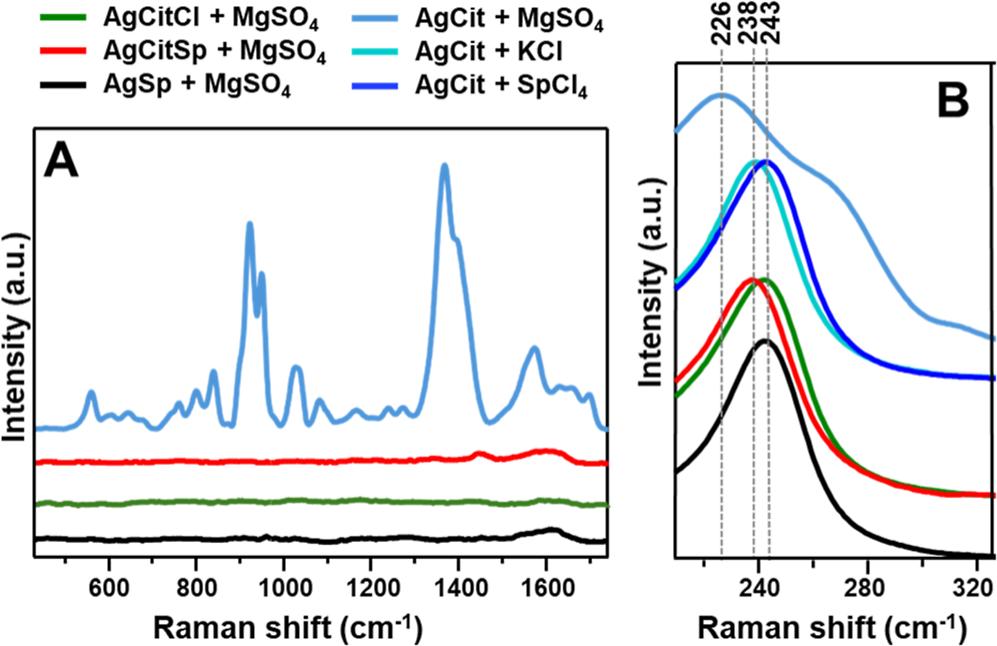
Background SERS spectra of each class of colloidal systems in the (A) higher and (B) lower wavenumber regions.

### Surface-Enhanced Raman Scattering Vibrational Signatures of Aptamers

Scheme 1 depicts the different colloidal systems investigated and the distinct aggregation pathways observed for cationic and anionic substrates. In these experiments, uApt or hApt was directly combined with the colloidal suspensions over a wide range of aptamer concentrations. The resulting mixtures were incubated overnight at room temperature, unless otherwise specified, to allow the colloid–aptamer interface to reach a stable adsorption equilibrium prior to SERS measurements. Under these conditions, the aptamer conformation at the interface is likely governed by the interplay between intrinsic folding dynamics and adsorption-induced constraints, resulting in a surface-stabilized conformational ensemble within the experimental time scale. In particular, the initially unfolded-enriched uApt ensemble should not be interpreted as remaining fully extended during incubation with the colloids. Upon adsorption, conformations accessible at early stages may become kinetically trapped, while large-scale rearrangements can be hindered by surface confinement and multivalent interactions with the nanoparticle interface. Therefore, the resulting SERS spectra of uApt are better described as arising from an adsorbed interfacial ensemble derived from an initially less-folded preparation state, rather than from a fully unfolded strand in bulk solution.

**Scheme 1:**
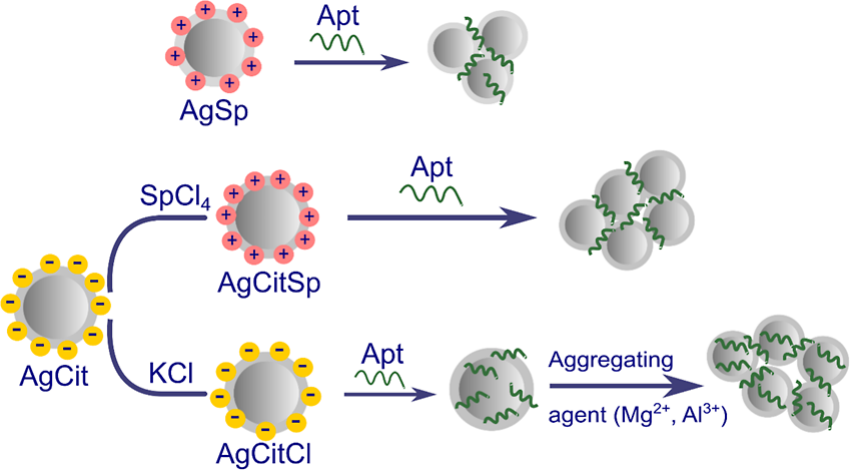
Schematic Representation of the Three Silver Colloidal Platforms Investigated: Intrinsically Cationic Spermine-Capped Silver Nanoparticles (AgSp), Post-Synthetically Spermine-Modified Citrate-Stabilized Silver Nanoparticles (AgCitSp), and Chloride-Modified Citrate-Stabilized Silver Nanoparticles Used as a Negatively Charged Control (AgCitCl)

For cationic colloids (AgSp and AgCitSp), aptamer addition induced immediate colloidal aggregation, driven by electrostatic interactions between the negatively charged phosphate backbone and the positively charged nanoparticle surface (Figure S3). Under these conditions, adsorption and junction formation occur over short time scales. In contrast, for negatively charged colloids (AgCitCl), aptamer addition alone does not induce aggregation, and nanoparticle clustering occurs only upon the addition of an external aggregating agent (MgSO_4_), resulting in the formation of metastable aggregates with time-dependent structural rearrangements and no control over nanoparticle assembly.^9,34^

It is worth noting that, under these aggregated colloidal conditions, the measured SERS signal is intrinsically dominated by a small subpopulation of molecules or, in the case of large biomolecules, by specific structural domains preferably located within the most plasmonically active interparticle junctions.^41,45^ Based on a previous quantitative modeling of direct SERS probing of a fully complementary 21-base DNA duplex (∼7 nm length, ∼2 nm diameter) adsorbed on cationic AgSp colloids, it was estimated that no more than approximately one duplex can be accommodated within the volume of the most efficient plasmonic hotspots.^15^ As a result, the registered SERS spectra are dominated by the contribution of this limited molecular fraction within hotspot regions, which, in principle, do not automatically constitute a weighted representation of the entire aptamer strands adsorbed across the entire metallic surface.

Figures S4–S13 present the full set of SERS spectra acquired in the concentration-dependent study for each nanoparticle system. In contrast to AgCitCl colloids, the concentration-dependent SERS spectra of both uApt and hApt on spermine-modified nanoparticles (AgSp and AgCitSp) exhibit invariant band positions across the investigated concentration range, while the spectral evolution is primarily manifested through subtle changes in relative band intensities (which are more evident for AgCitSp than for AgSp). On the other hand, for the 70 nm silver colloids (AgCitSp and AgCitCl), concentration-dependent spectra of both uApt and hApt were successfully collected under excitation at 514 and 785 nm, respectively. In contrast, for the smaller AgSp nanoparticles, measurable SERS signals were obtained under 514 nm excitation, whereas no appreciable signals were detected under near-infrared excitation, which is consistent with the limited ability of these small particles to generate an efficient electromagnetic enhancement at longer wavelengths. Therefore, to enable a direct comparison across all colloidal systems, subsequent spectral analysis was restricted to the SERS data acquired under 514 nm excitation.

Figure 4 shows the representative SERS spectra of uApt and hApt on the different colloidal platforms, selected at comparable analyte concentrations and signal qualities to enable an initial qualitative comparison of their spectral features. The vibrational band assignments of the main NA features discussed in this study are summarized in Table S2.

**Figure 4:**
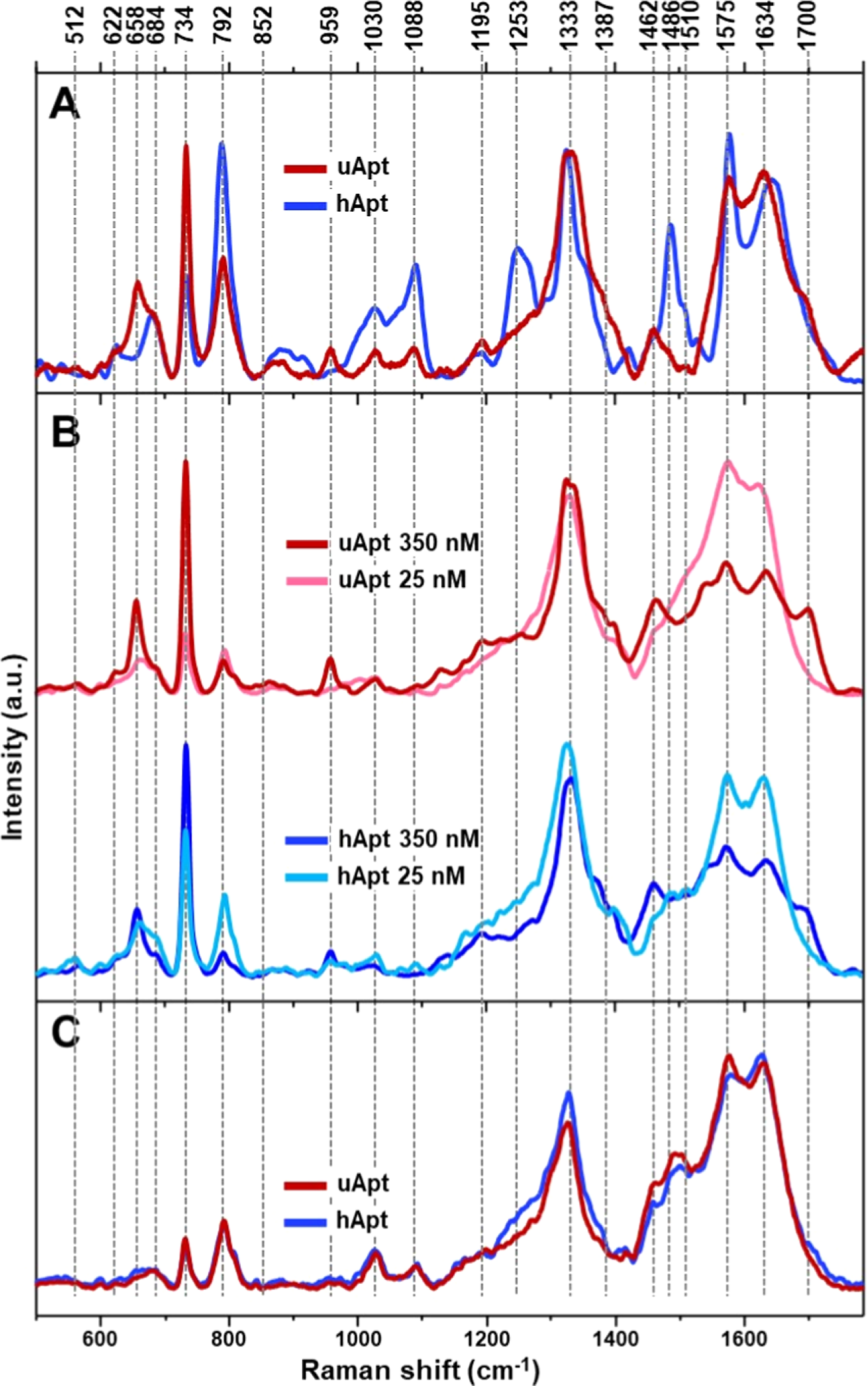
Representative SERS spectra of uApt and hApt adsorbed on different colloids. The intensities of all the spectra were adjusted for visual comparison. (A) AgSp colloids; aptamer concentration: 350 nM. (B) AgCitCl colloids aggregated with MgSO_4_ at aptamer concentrations of 25 nM and 350 nM. (C) AgCitSp; aptamer concentration: 25 nM.

#### Aptamers on AgSp Colloids

In the case of AgSp (Figure 4A), the spectral changes observed upon transitioning from the predominantly extended conformation to the folded hairpin structure closely mirror those reported in the literature for the hybridization of complementary single-stranded DNA into the corresponding duplex on positively charged AgSp colloids.^9,31^ Structural rearrangements associated with base-pairing are reflected in the spectra through both significant band shifts and marked redistributions of relative intensities.^9^

In the low-wavenumber region, we highlight the red shift of the adenine (A) ring breathing bands centered at ∼735 cm^–1^, together with a more pronounced red shift of the band located around 790 cm^–1^, commonly assigned to cytosine/thymine (C + T) ring breathing vibrations. Cytosine has been shown to contribute more strongly than thymine to this vibrational mode on AgSp colloids.^46^ Moreover, in the present aptamer sequence, T bases are present in a limited number (three), whereas C residues are significantly more abundant (11). Accordingly, the band at ∼790 cm^–1^ can be primarily attributed to C scattering, with only a marginal contribution from T.

In the 600–700 cm^–1^ region, the SERS response is dominated by guanine (G) ring breathing modes, which are well-known to be highly sensitive to local stacking interactions, hydrogen bonding, and electrostatic environment.^47,48^ Particularly, features around ∼622, ∼658, and ∼686 cm^–1^ have been widely used in Raman studies as duplex conformational markers associated with Z-, A-, and B-type DNA environments, respectively.^47,48^ In the case of hApt, the G band near ∼686 cm^–1^ clearly dominates this spectral region, suggesting that the folded aptamer retains a predominantly duplex-like local organization upon surface binding. In contrast, for uApt, multiple guanine-associated features coexist in the 600–700 cm^–1^ region, with the band near ∼658 cm^–1^ being the most intense. This spectral profile, characterized by broadened and redistributed guanine bands, is indicative of weakly stacked or disordered nucleobases, which is consistent with the higher conformational disorder and flexibility of the unfolded chain.

Simultaneously, the broad composite carbonyl stretching band centered at approximately 1635 cm^–1^ undergoes a marked blue shift. This band is primarily attributed to the C=O stretching vibrations of pyrimidine bases (T and C), representing a well-established marker of vibrational coupling associated with Watson–Crick base pairing.^9,48,49^ A major reshaping of the spectral profile is also observed in the 1200–1500 cm^–1^ region, which is dominated by complex, overlapping in-plane ring stretching and bending vibrations of the nucleobases.

Finally, the spectral envelope in the 900–1100 cm^–1^ region comprises vibrational features primarily associated with the NA backbone, such as the band at approximately 1030 cm^–1^, commonly attributed to coupled ribose and cytosine vibrations, as well as the intense band at ∼1089 cm^–1^, assigned to the symmetric PO_2_^–^ stretching mode of the phosphate backbone. The remarkable intensity of this phosphate-related band, despite its significantly lower Raman cross-section compared to nucleobase vibrations,^50^ indicates the close proximity of the phosphate groups to the metallic surface.

Overall, the ensemble of spectral changes is consistent with a cooperative reorganization of base stacking and base-pairing interactions, suggesting that the duplex-like character of the hApt conformation, relative to the predominantly extended uApt, is largely preserved upon the formation of the surface-bound complex.

However, several systematic deviations from the behavior of fully hybridized 21 base pair duplex DNA are also evident,^9,31^ reflecting the heterogeneous distribution of paired and unpaired nucleobases inherent to the hairpin architecture. First, in the case of the adenine ring breathing mode, the shift associated with the uApt-to-hApt transition is limited to approximately 2 cm^–1^, in contrast to the larger shifts of about 4–5 cm^–1^ typically reported for full duplexes on AgSp colloids.^9,31^ Conversely, the shift of the cytosine-ring breathing band reaches ∼5 cm^–1^, closely approaching the values of ∼6 cm^–1^ observed for fully complementary duplexes.^9,31^ This behavior is consistent with the structural organization of the hairpin aptamer, in which adenine residues are predominantly located within the apical loop and therefore remain largely unpaired, whereas the majority of cytosine bases are engaged in Watson–Crick pairing with guanine within the stem region. Second, we observe a marked redistribution of the relative intensities of the ring-breathing mode bands, most notably a drastic inversion of the A/C intensity ratio upon folding. In sharp contrast, we have previously shown that for ssDNA-to-dsDNA hybridization on positively charged silver colloids,^31^ the relative intensities of the A/C ring breathing modes in the SERS spectra of the duplex can be reasonably approximated as a weighted average of the corresponding ratios obtained from the two individual single-stranded constituents.

Previous normal Raman studies have demonstrated that spermine interacts more efficiently with double-stranded DNA than with single-stranded sequences, owing to the higher rigidity and more regular spatial organization of phosphate groups in duplex structures, particularly in GC-rich regions.^51–54^ Consistent with this picture, duplex DNA has been shown to promote more efficient plasmonic hotspot formation on cationic AgSp colloids than single-stranded DNA, a behavior that we previously correlated with the markedly lower concentrations required for duplexes to yield SERS spectra of comparable intensity.^15,31,34^

Within this framework, the folded hairpin can be regarded as a heterogeneous structure comprising a local duplex-like C-rich stem and an A-rich loop. Therefore, preferential electrostatic coupling between surface-confined spermine and the stem region is expected, leading to a more efficient trapping of the stem within the most plasmonically active interparticle junctions. Consequently, the SERS response of the hairpin should be skewed toward the vibrational contributions associated with cytosine, whereas the contribution of adenine-rich loop regions is comparatively attenuated. This selective enhancement appears to be directly reflected in the experimentally observed drastic reduction in the A/C intensity ratio upon folding, providing a clear spectroscopic signature of the preferential involvement of the stem region in the SERS enhancement.

It is worth stressing that no systematic increase in the guanine ring breathing band intensity is observed upon folding. In principle, this does not contradict the preferential localization of the duplex-like stem within the hot spots but likely reflects the intrinsic spectral complexity of guanine.^9^ Indeed, when comparing uApt and hApt on AgSp, where guanine-associated SERS bands differ markedly in shape and relative intensity, the guanine response is dominated by conformational reorganization. Consequently, guanine bands cannot be used as reliable reporters of segment-specific localization in such comparisons.

#### Aptamers on AgCitCl Colloids

Several studies have demonstrated that oligonucleotides and individual nucleosides can directly interact with negatively charged silver colloids through the localized chemisorption of exposed nucleobases.^55,56^ Such interactions mainly occur via the exocyclic amine moiety or deprotonated heterocyclic nitrogen, while the phosphate backbone remains largely decoupled from the metal surface.^55,56^ This adsorption mechanism accounts for the dominance of vibrational modes associated with the aromatic nucleobases in SERS spectra, compared to those of the sugar and phosphate residues, which is also clearly reflected in the spectra of both uApt and hApt (Figure 4B). Notably, adenosine has been consistently reported as the nucleoside with the highest affinity for binding silver and gold, mainly via the N7-nitrogen atom. This interaction is highly sensitive to the surface properties of the metal substrate and to surface coverage with lateral packing effects modulating the preferred orientation of adenosine at the interface.^56,57^

Compared to spermine-coated colloids, the concentration-dependent SERS response on AgCitCl displays a more complex spectral evolution, characterized not only by variations in relative band intensities but also by the progressive emergence and reshaping of spectral features (Figures S8 and S9). In contrast, the spectral differences between uApt and hApt at comparable concentrations on AgCitCl are minor. This behavior is consistent with our previous reports on MgSO_4_-aggregated negatively charged silver colloids, where short single-stranded and double-stranded DNAs yield almost identical SERS spectral profiles.^34^ To qualitatively assess these effects, representative SERS spectra of uApt and hApt recorded at low (25 nM) and high (350 nM) aptamer concentrations are shown in Figure 4B.

Within the low-wavenumber region (500–690 cm^–1^), the adenine ring breathing mode remains essentially invariant in position as the aptamer concentration increases, whereas the cytosine/thymine ring breathing band exhibits only a minor shift of approximately 1 cm^–1^. In contrast, a progressive redistribution of relative intensities is observed among guanine-associated bands around ∼658 and ∼686 cm^–1^ upon increasing aptamer concentration. Since the SERS spectra of the extended and hairpin aptamer conformations are nearly identical when measured at the same concentration, this intensity redistribution cannot be attributed to base pairing effects. Rather, it should reflect a rearrangement of guanine conformational populations and local microenvironment at the metal interface.

In the backbone-associated spectral region, we also observe a clear inversion in the relative intensities of the ribose–phosphate stretching band around 955–960 cm^–1^, a conformational marker sensitive to the geometry and ordering of the NA backbone,^49^ and the ribose/cytosine band at approximately 1030 cm^–1^. Notably, the PO_2_^–^ symmetric stretching band at ∼1089 cm^–1^ appears strongly attenuated, in sharp contrast to its pronounced intensity on spermine-modified colloids.

As previously highlighted, the 1140–1400 cm^–1^ region is dominated by a broad composite envelope arising from the superposition of multiple in-plane ring stretching and bending modes of all nucleobases. Given the strong spectral overlapping and the intrinsically mixed character of the contributing vibrations, a detailed band-by-band analysis is not attempted here. Nevertheless, the overall band shape evolves systematically with increasing aptamer concentration, indicating significant changes in the relative weighting of the underlying vibrational contributions.

Finally, in the high-wavenumber region, the band at approximately 1460 cm^–1^, mainly associated with C + T ring modes, together with the weak high-frequency contribution near 1700–1720 cm^–1^ corresponding to the guanine C=O stretching mode, increases in relative intensity compared to the features around 1575 cm^–1^, assigned to mixed A + G ring modes, and the band at ∼1634 cm^–1^, attributed to T + C carbonyl stretching vibrations.

Taken together, the spectral dominance of nucleobase modes and the attenuation of the phosphate band strongly indicate that nucleobase–silver interactions largely govern the interfacial configuration, although a contribution from thiol-mediated anchoring cannot be excluded. Furthermore, the close similarity between the SERS spectra of uApt and hApt at comparable concentrations, together with the much larger spectral changes observed upon increasing aptamer concentration, suggests that the dominant spectral evolution is not primarily associated with discrete folding (i.e., hairpin vs extended conformation) of the NA within the surface complex. Accordingly, the most plausible interpretation is that the observed changes are likely associated with surface-coverage-dependent rearrangements of the adsorbed chain, driven by lateral packing and aggregation-induced crowding effects.

#### Aptamers on AgCitSp Colloids

In the case of spermine-modified citrate colloids (AgCitSp, Figure 4C), the SERS response exhibits an approximate intermediate behavior between that observed for AgSp and for negatively charged AgCitCl colloids. On the one hand, upon increasing aptamer concentration, the spectra do not display the pronounced spectral reshaping characteristic of AgCitCl systems, but mainly gradual variations in relative band intensities, particularly in the regions associated with A and C ring breathing modes (Figures S6 and S7). On the other hand, the clear and systematic spectral differences between uApt and hApt observed on AgSp are no longer evident, with the two conformational states yielding closely similar spectral profiles at comparable concentrations. At the same time, the phosphate-related band at ∼1089 cm^–1^ remains clearly visible on AgCitSp, although its relative intensity with respect to the remaining spectral features is markedly reduced compared to AgSp. Moreover, in the 1140–1400 cm^–1^ region, the overall spectral profile shows clear similarities to those observed for uApt on AgSp and for both uApt and hApt on AgCitCl, while, in the high-wavenumber region (>1400 cm^–1^), it appears qualitatively intermediate between that observed for aptamers on AgCitCl at low and high concentrations.

Overall, these observations indicate that AgSp and AgCitSp stabilize distinct interfacial DNA complexes despite both being modified with spermine. This indicates that the presence of a “spermine layer” does not necessarily define a unique surface state, but rather a family of interfacial configurations whose properties depend on multiple, interrelated physical and chemical parameters.

While differences in nanoparticle diameter between AgSp and AgCit are expected to influence hotspot geometry and electromagnetic field confinement, curvature effects on DNA adsorption geometry are likely modest at the scale of the aptamer and should not, by themselves, account for the pronounced differences observed in the SERS response.

In this respect, the substantially different bulk spermine-to-surface-area ratios employed for AgSp and AgCitSp could promote distinct adsorption regimes and interfacial organizations of the polyamine layer. Such differences may translate into positively charged shells with different packing, hydration, and structural organization which can critically affect DNA-surface proximity and binding motifs within the surface complex.

Importantly, the chemical nature of the underlying silver surface itself is also strongly conditioned by the synthetic route. For instance, our previous comparison between analogous AgCitCl colloids and silver nanoparticles synthesized using hydroxylamine hydrochloride demonstrated that formally “chloride-coated” nanoparticles can nevertheless exhibit markedly different SERS responses toward short oligonucleotides composed of a single nucleobase type.^34^ These observations indicate that the mere presence of chloride is insufficient to uniquely define the effective surface chemistry experienced by the adsorbed molecule. Consistent with established SERS models, halide ions, particularly chloride, actively participate during nanoparticle growth by stabilizing low-coordination Ag sites and adatom-like species that are widely regarded as key contributors to SERS activity.^43^ Within this framework, the distinct behavior observed for spermine-coated AgSp and AgCitSp may reflect differences in the intrinsic surface chemistry imprinted during nanoparticle formation, which in turn could influence the adsorption configuration and interfacial organization of spermine and, consequently, its interaction with the aptamer. Interestingly, the same study also showed that chloride-modified colloids with substantially different nanoparticle diameters (∼30 vs ∼70 nm) can yield highly similar SERS spectra for structurally complex NAs, whereas significant spectral differences emerge when chloride is replaced by iodide on nanoparticles of comparable size.^34^ Collectively, these observations suggest that the resulting adsorption behavior and consequently the observed vibrational pattern are likely more strongly influenced by the specific interplay between nucleic-acid structural complexity and nanoparticle surface chemistry than the nanoparticle size.

Finally, it is important to note that neither salt-aging protocols nor freeze–thaw strategies were employed to promote extensive thiol-mediated anchoring to the nanoparticle surface.^30,58^ Consistently, none of the SERS spectra of DNA recorded on the different colloidal substrates exhibit the characteristic vibrational signatures typically reported for densely packed alkanethiol- or PEG-based surface modifications on silver nanoparticles.^59^

Moreover, control experiments performed after TCEP treatment of the thiolated aptamer did not reveal major alterations in the overall SERS fingerprint (Figure S14). Since TCEP was not removed after reduction, these experiments should be interpreted as reductive-treatment controls performed in the presence of residual low-micromolar TCEP, rather than as a strict isolation of free-thiol effects. However, this residual TCEP concentration is approximately 2 orders of magnitude lower than that reported to induce significant TCEP-mediated effects on DNA adsorption at plasmonic nanoparticle surfaces.^60^ Thus, although minor contributions from thiol-mediated interactions or residual TCEP cannot be fully excluded, they do not appear to dominate the observed vibrational response under the investigated conditions.

### Concentration-Dependent Trends of Ratiometric Surface-Enhanced Raman Scattering Markers

Based on the vibrational assignments and qualitative spectral analysis discussed above, the following SERS intensity ratios were used as ratiometric indicators to support the proposed hypotheses. It should be noted that these ratiometric markers are used here as qualitative descriptors of the spectral evolution and as internal support for comparing the different colloidal systems under matched experimental conditions. They are not intended to define a statistically validated concentration-dependent model or an analytical calibration.

(1) Ratios between nucleobase ring breathing modes for segment selection (A/C and A/G). The ratios between the ring breathing modes of adenine (∼735 cm^–1^), cytosine (∼792 cm^–1^), and guanine (∼685 cm^–1^) were introduced to support the hypothesis of preferential involvement of distinct aptamer segments within SERS-active hotspots, as inferred from the qualitative redistribution of nucleobase band intensities. In the case of hApt, the intrinsic asymmetric base composition (GC-rich stem, vs A-rich apical loop) provides a clear structural rationale for interpreting A/C and A/G ratios as indirect probes of stem versus loop contribution. For the extended uApt, the same ratios instead support preferential involvement of internal versus terminal sequence regions, consistent with the heterogeneous base distribution of the unfolded sequence, which contains A-rich motifs flanked by GC-rich segments, particularly toward the 3′ terminus. Within this framework, A/C ratios were identified as the most reliable indicators, as they rely on well-resolved vibrational bands with limited sensitivity to orientation-specific effects. In contrast, A/G ratios were treated only as secondary, qualitative support, owing to the intrinsic spectral complexity and conformational sensitivity of guanine-associated features.
(2) Ratios between nucleobase and phosphate backbone modes for surface proximity trends (A/P, C/P). The second category includes ratios between A and C ring breathing modes and the phosphate backbone vibration at ∼1089 cm^–1^ (A/P and C/P). The symmetric phosphate stretching mode is comparatively insensitive to the intrinsic DNA conformation in the bulk (e.g., hairpin versus extended, A- versus B-like) while remaining sensitive to distance and orientation effects at the metal interface. Accordingly, these ratios were introduced to support qualitative observations regarding the relative proximity of nucleobases versus the backbone to the metal surface.
(3) Ratios between guanine-associated modes for local rearrangement (G/G). The third category consists of ratios between guanine-associated modes (∼685 vs ∼658 cm^–1^), which were used exclusively to support hypotheses of local conformational rearrangements involving guanine bases, previously inferred from qualitative spectral reshaping. However, given the intrinsic broadening of guanine-associated SERS bands, concentration-dependent variations of the G/G ratio must be interpreted with caution.

For hApt adsorbed on AgSp, all classes of ratiometric markers (Figures 5 and S15) exhibit a very weak or null dependence on NA concentration, suggesting that variations in surface coverage do not lead to significant measurable changes in either the structural organization of the aptamer within the surface complex or its spatial distribution within SERS-active hotspots (within the sensitivity limits of the ratiometric approach employed). In contrast, for the extended aptamer, a subtle concentration-dependent variation of A/C, A/G, A/P, and C/P ratios is observed with increasing NA concentration. A closely analogous behavior is observed for both aptamer forms on AgCitSp, with ratiometric markers involving adenine, cytosine, and phosphate groups (A/C, A/G, A/P, and C/P) displaying trends that closely mirror those observed on AgSp colloids except for the guanine-based ratios (G/G).

**Figure 5:**
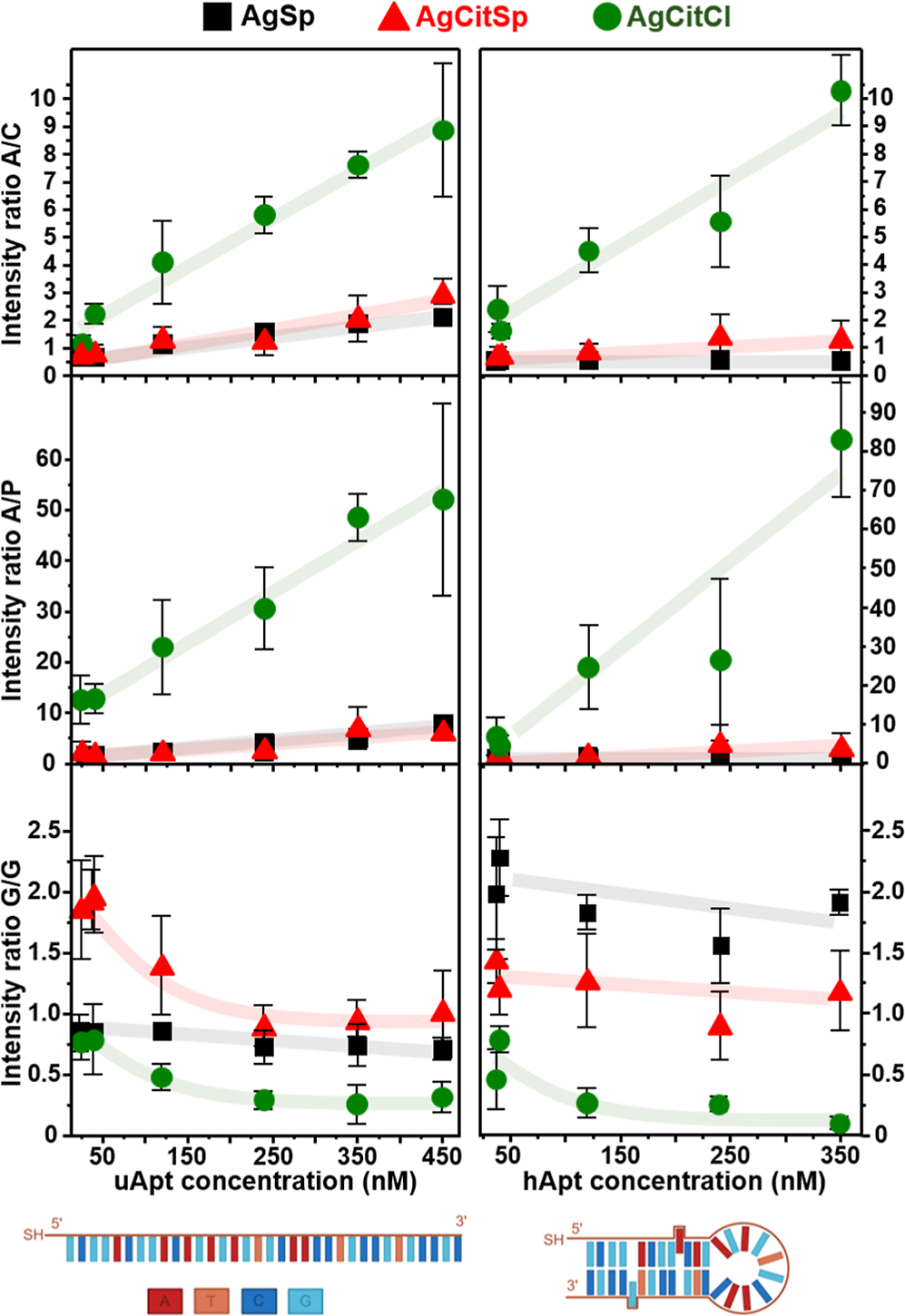
Aptamer concentration dependence (unfolded vs hairpin) of the ratiometric markers: A/C (ring breathing modes adenine vs cytosine), A/P (ring breathing mode of adenine versus phosphate symmetric stretching), and G/G (guanine stretching modes ∼685 vs ∼658 cm^–1^) for the three colloidal platforms investigated (AgSp, AgCitSp, and AgCitCl). Solid lines are included as visual guides. Data points represent mean values obtained from the two independent experimental series (error bars indicate the standard deviation calculated from the corresponding values).

These observations are consistent with previous reports investigating the adsorption of single-stranded DNA (ssDNA) chains of comparable length on amino-functionalized, positively charged surfaces.^61–63^ While ssDNA is generally found to adopt a predominantly flattened adsorption geometry driven by strong electrostatic coupling between the phosphate backbone and protonated surface amino groups, increased steric constraints and local mismatches between surface charge density and backbone charge can result in a non-negligible fraction of chains remaining only partially flattened. In such cases, segments of the chain may protrude into solution, giving rise to a broader distribution of adsorption geometries. Accordingly, the distinct ratiometric behavior observed for hApt and uApt on spermine-coated silver colloids can be rationalized in terms of differences in their conformational plasticity. In contrast to the structurally flexible extended aptamer, the intrinsically rigid and preorganized hairpin displays limited susceptibility to changes in surface coverage. On the other hand, the drastic differences observed in guanine-based G/G ratios between AgSp and AgCitSp colloids exemplify the distinct conformational nature of the aptamer–spermine surface complex on the two substrates.

Finally, a markedly different behavior is observed for both aptamer forms adsorbed on AgCitCl colloids and aggregated by the addition of MgSO_4_. At low surface coverage, the ratiometric values approach those measured for uApt on AgSp, suggesting a similarly flattened adsorption geometry despite the fundamentally different interfacial interaction mechanism, as independently evidenced by the distinct SERS spectral profiles. However, as surface coverage increases, a pronounced relative enhancement of adenine-associated contributions with respect to both cytosine and the phosphate backbone emerges, while the G/G ratio progressively decreases until reaching a plateau.

When the aptamer concentration is normalized by the estimated total metallic surface area (Figure S16), the distinct concentration-dependent trends observed for AgSp, AgCitSp, and AgCitCl are preserved, indicating that the differences cannot be simply attributed to the different nanoparticle surface areas available in each colloidal system.

Overall, these ratiometric tendencies support a markedly higher conformational flexibility of both uApt and hApt on negatively charged colloids, indicating that coverage-dependent modulation of the adsorption geometry, rather than the intrinsic folding state of the DNA, appears to dominate the response on these metallic surfaces. In this framework, the progressive enhancement of adenine-associated bands with increasing surface coverage is consistent with the notion that A bases, which are known to display a relatively higher affinity for silver surfaces compared to other nucleobases, become increasingly involved in metal-nucleobase interactions under crowded conditions at the expense of CG-rich regions. Consequently, surface-induced reorganization possibly favors configurations in which A-rich segments of the sequence are positioned closer to the metal surface and/or more efficiently trapped within the most plasmonically active interparticle gaps. This interpretation is further supported by the incubation-time-dependent spectral evolution observed for AgCitCl, which indicates progressive interfacial rearrangement of the adsorbed aptamer toward an equilibrium configuration.

### The Role of the Aggregating Agent and Incubation Time for Negatively Charged Colloids

SERS measurements on AgCitCl were performed following a two-step protocol in which the aptamer was first equilibrated overnight with the colloidal suspension, and nanoparticle aggregation was subsequently induced by the addition of magnesium sulfate.

The final Mg^2+^ concentration in the sample (35 mM) has been reported to be sufficiently high to substantially perturb the electrostatic environment of NA in NA–cation systems under homogeneous aqueous systems.^64,65^ Indeed, it is well established that multivalent cations of different nature interact with DNA through distinct binding modes, giving rise to ion-specific DNA–cation complexes with different structural and electrostatic characteristics. For instance, Mg^2+^ predominantly contributes through partial electrostatic screening,^66–68^ while trivalent ions such as Al^3+^ are known to engage in stronger and more specific ion–phosphate interactions, which may induce additional structural rearrangements or even precipitation under near-physiological conditions.^69,70^ These ion-specific effects are also reflected in the SERS fingerprint of the surface complex. As shown in Figure S17, replacing MgSO_4_ with Al­(NO_3_)_3_, while maintaining the same overnight incubation protocol, leads to a pronounced reshaping of the vibrational profile for both aptamer forms. Although the local ionic environment at the metal–liquid interface is expected to differ from bulk solution conditions, these results indicate that the nature of the aggregating cation plays a direct role in defining the spectroscopic signature of the surface-bound complex.

A second variable concerns the incubation time prior to aggregation, keeping MgSO_4_ as the aggregating agent. When the preaggregation incubation time was shortened from overnight to 10 min, the spectral profile underwent a marked reshaping (Figure 6, red curve). Interestingly, this spectrum is substantially more similar to that obtained when uApt was first incubated overnight with MgSO_4_ prior to the addition of the colloids (Figure 6, purple curve) than to the spectrum recorded after overnight equilibration on AgCitCl (Figure 4B, uApt 25 nM). This is further corroborated by the analysis of the ratiometric ratios (Figure S18). It is also worth noting that, compared to the overnight-equilibrated AgCitCl sample, both SERS spectra in Figure 6 exhibit a major enhancement of several ring modes mainly attributed to guanine (∼512, 1195, 1387, and 1510 cm^–1^).^9^ The ribose-related band near ∼852 cm^–1^ is also concomitantly intensified, whereas the phosphate vibration at ∼1089 cm^–1^ remains consistently weak under all preparation conditions.

**Figure 6:**
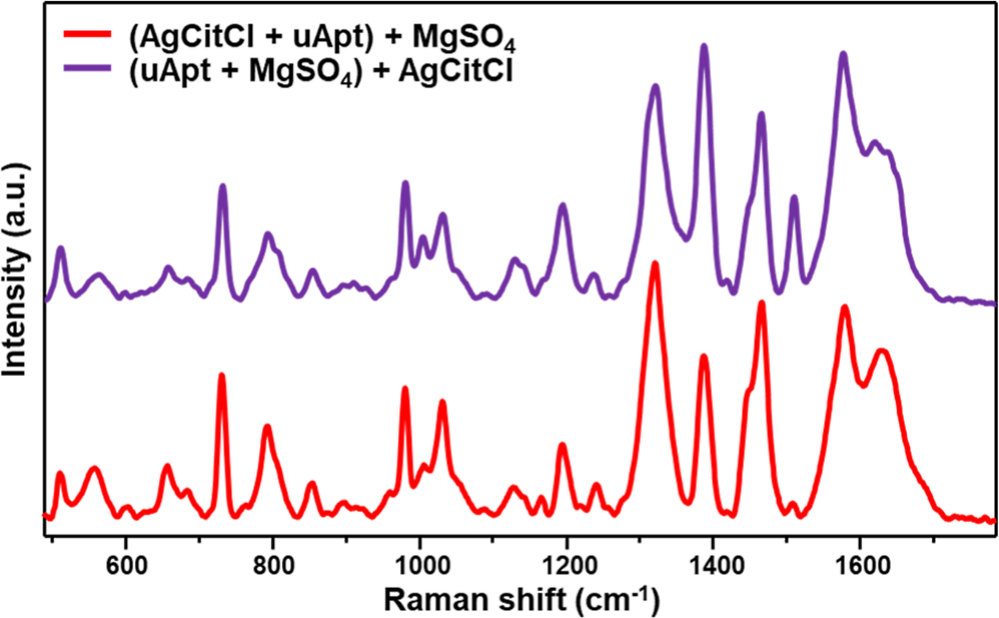
SERS spectra of uApt (25 nM) recorded on AgCitCl colloids aggregated with MgSO_4_ under different preparation protocols. (Red curve) uApt was incubated with the colloidal suspension for 10 min prior to the addition of the aggregating agent. (Purple curve) uApt was first incubated overnight with MgSO_4_, and the colloids were subsequently added, leading to rapid nanoparticle aggregation.

In contrast, in AgSp colloids, aptamer adsorption and nanoparticle aggregation occur concomitantly upon the addition of an analyte. The system rapidly forms DNA-mediated nanoparticle clusters, followed by a short equilibration phase during which subtle fluctuations of the relative intensities are progressively averaged out.^46^ After this initial stage, the SERS response remains essentially invariant over time, indicating that a stable interfacial configuration is rapidly established. This behavior is fundamentally different from that observed in negatively charged colloids (AgCitCl), where adsorption and aggregation are decoupled processes. In this case, the aptamer appears to undergo a relatively slow equilibration at the nanoparticle surface prior to salt-induced aggregation, the extent of which strongly influences the resulting SERS fingerprint. Consequently, an insufficient incubation time before salt-induced aggregation leads to differences in surface-complex configurations and spectral responses.

## Conclusions

In this work, we have systematically investigated the direct SERS fingerprinting of a thiolated aptamer adsorbed on silver colloids with distinct surface properties. Comparative analysis indicates that, under the investigated conditions, nanoparticle surface chemistry primarily defines the dominant adsorption regime by modulating the balance between backbone-mediated electrostatic interactions and nucleobase–metal coordination. Consequently, the same aptamer sequence gives rise to distinct spectral profiles depending on the interfacial electrostatic environment and surface functionality.

In spermine-modified colloids, electrostatic screening promotes a more constrained adsorption geometry, leading to stable and reproducible vibrational fingerprints in which segment-specific contributions can be interpreted more reliably. Notably, only AgSp colloids, where spermine is incorporated during nanoparticle synthesis rather than introduced by post-synthetic surface modification, enable clear spectral discrimination between the unfolded and hairpin forms of the aptamer. This observation indicates that the specific interfacial organization generated during nanoparticle formation appears to preserve folding-dependent differences more effectively within the surface complexes.

By contrast, in negatively charged chloride-modified systems, the SERS response is extensively influenced by coverage- and time-dependent rearrangements of the adsorbed chains, reflecting substantial conformational plasticity and progressive surface-induced reorganization toward equilibrium configurations. In particular, the pronounced incubation time dependence observed for AgCitCl prior to aggregation highlights the role of slow interfacial equilibration processes in defining the final SERS fingerprint of the surface-bound complex. Under these conditions, lateral packing and crowding effects outweigh the intrinsic folding state of the NA in determining the observed spectral evolution. At the same time, this pronounced sensitivity is accompanied by concentration-dependent changes in selected ratiometric markers, particularly adenine-based ratios, such as A/C and A/P. These markers provide qualitative support for the coverage-dependent spectral evolution across the investigated colloidal systems. In this regime, ratiometric analysis offers a useful internal normalization approach to compare relative spectral changes while reducing the impact of fluctuations in the absolute SERS intensity. However, it should not be interpreted here as a statistically validated quantitative model. Conversely, this approach is less informative for spermine-stabilized systems, where the spectral profile remains largely concentration invariant.

Overall, these findings suggest that direct SERS probing of aptamers in colloidal suspension yields a vibrational signature that emerges from the specific interplay between NA structural complexity and nanoparticle interfacial chemistry, the latter being determined not only by surface composition itself but also by the specific mode through which surface-active species are incorporated during nanoparticle formation or post-synthetic modification. Careful control and characterization of nanoparticle surface chemistry are therefore essential for extracting meaningful structural information and for the rational design of SERS-based NA sensing platforms. At the same time, the distinct adsorption regimes imposed by different colloidal substrates suggest that complementary spectroscopic information can be obtained under controlled surface-chemistry conditions.

## Supplementary Material



## Notes

Absorption spectra of uApt and hApt solutions, nanoparticle size characterization, vibrational band assignments, extinction spectra of colloids after overnight incubation with uApt, normalized SERS spectra at different aptamer concentrations, SERS spectra of hApt before and after TCEP treatment, aptamer concentration dependence of ratiometric markers, surface area normalized aptamer concentration dependence of the ratiometric markers, SERS spectra of aptamer-adsorbed AgCitCl colloids aggregated with Al­(NO_3_)_3_, and ratiometric analysis of different preparation protocols of AgCitCl and aptamer colloids (DOCX)
