# Supplementary material for: Surface Chemistry-Driven Surface-Enhanced Raman Scattering Fingerprinting of Aptamers on Silver Colloids
Source: ACS Appl Opt Mater. 2026 Jul 9;4(7):2013–27. doi: 10.1021/acsaom.6c00151 (PMC13435865; doi:10.1021/acsaom.6c00151)
Supplement: Supplementary file 1 [file ot6c00151_si_001.docx]

Supporting Information

Surface Chemistry-Driven SERS Fingerprinting of Aptamers on Silver Colloids

Sara Pandolfi,^1^ Elisabetta Venuti,^1^Erica Locatelli,^1^ Xavier Mateos,^2^ Nicolas Pazos-Perez,^3,*^ Tommaso Salzillo,^1,*^ Luca Guerrini.^3,*^

^1^ University of Bologna, Department of Industrial Chemistry “Toso Montanari”, via Piero Gobetti 85, 40129 Bologna, Italy.

^2^ University Rovira i Virgili, Physics and Crystallography of Materials research group, FiCMA, Marcel∙lí Domingo 1, 43007 Tarragona, Spain.

^3^ University Rovira i Virgili, Department of Physical and Inorganic Chemistry, Marcel∙lí Domingo 1, 43007 Tarragona, Spain.

Corresponding Authors

*Nicolas Pazos-Perez - University Rovira i Virgili, Department of Physical and Inorganic Chemistry, Marcel∙lí Domingo 1, 43007 Tarragona, Spain. Email: nicolas.pazos@urv.cat

*Tommaso Salzillo - University of Bologna, Department of Industrial Chemistry “Toso Montanari”, via Piero Gobetti 85, 40129 Bologna, Italy. Email: tommaso.salzillo@unibo.it

*Luca Guerrini - University Rovira i Virgili, Department of Physical and Inorganic Chemistry, Marcel∙lí Domingo 1, 43007 Tarragona, Spain. Email: luca.guerrini@urv.cat

**Table of Contents**

Absorption spectra of uApt and hApt	S3

Nanoparticle size characterization	S4

Vibrational bands assignments	S5

Extinction spectra of colloids after overnight incubation with uApt	S6

Normalized SERS spectra at different aptamer concentrations	S7

SERS spectra of hApt before and after TCEP treatment	S17

Aptamer concentration dependence of ratiometric markers	S18

Surface area normalized aptamer concentration dependence of the ratiometric markers	S19

SERS spectra of aptamer adsorbed on AgCitCl aggregated with Al(NO_3_)_3_	S20

Ratiometric analysis of different preparation protocols of AgCitCl and aptamer colloids	S21

References	S22

**Figure S1**. UV–Vis spectra for equimolar uApt and hApt (2.5 μM) under their respective preparation conditions, namely uApt after thermal shock in Milli-Q water and hApt after annealing in PBS. Under these conditions, hApt exhibited a lower absorbance in the 250–270 nm region, which is qualitatively consistent with increased base stacking and/or intramolecular organization.

Nanoparticle sizes were determined from TEM images using ImageJ. The nanoparticles show a size distribution of 70 nm for AgCit and 30 nm for AgSp, as shown in Figure S2. Data correlating size and extinction coefficient of silver nanoparticles, from the work of Yguerabide,^1^ were plotted and fitted by Origin using the Chapman exponential model. From the fitted model the extinction coefficient was inferred knowing the size of the particles, measured by TEM. By means of Lambert Beer law, $A=c \epsilon L$, where *A* is the measured absorbance, *c* is the concentration, 𝜖 is the extinction coefficient, and L the path length of the measured sample, which depends on the cuvette used, 1 cm in our case, the concentration of the nanoparticles was calculated, as reported in Table S1.

**Table S1**. Nanoparticles used for SERS and their characterization data: the maximum absorbance was measured by UV–Vis spectroscopy, the size was determined by analysis of TEM images, the surface charge (ζ-potential) was measured by electrophoretic light scattering, and the extinction coefficient and concentration were inferred as described above.

**Figure S2**. (a, c) TEM images picture of AgSp in light blue and AgCit in green; (b, d) particle size distribution inferred by TEM images using ImageJ, of AgSp in light blue and AgCit in green; (e) fit of the data obtained from literature of Ag NP extinction coefficients varying with NP size. The fit function and parameters were eventually used to infer the extinction coefficient of AgSp and AgCit, knowing their size.

| **Type of NP** | **Max 𝜆_abs_ (nm)** | **Size (nm)** | **Surface charge (mV)** | **Extinction coefficient**  **(M^-1^cm^-1^)** | **[NP] (nM)** |
| --- | --- | --- | --- | --- | --- |
| *AgSp* | 393 | 30 ± 10 | + 39 | 1.7 x 10^10^ | 0.3 |
| *AgCitSp* | 446 | 70 ± 10 | + 25 | 7.1 x 10^10^ | 0.03 |
| *AgCitCl* | 446 | 70 ± 10 | - 40 | 7.1 x 10^10^ | 0.04 |

| AgSp | | AgCitSp | | AgCitCl+ Mg^2+^ | | AgCitCl+ Al^3+^ | |  |  |
| --- | --- | --- | --- | --- | --- | --- | --- | --- | --- |
| uApt | hApt | uApt | hApt | uApt | hApt | uApt | hApt | Band assignment ^2^ | Spectral region |
| 601 | 601 | 601 | 601 | 602 | 602 | 593 | 593 | C, ring ^3^ | Ring bending modes |
| 626 | 623 | 624 | 624 | 624 | 624 | 626 | 626 | G, ring ^3^ |  |
| 658 | - | 655 | 655 | 656 | 662 | - | - | G, ring br. |  |
| 685 | 680 | 685 | 685 | 685 | 685 | 685 | 685 | G, ring br. |  |
| 734 | 730 | 733 | 732 | 733 | 734 | 730 | 730 | A, ring br. |  |
| 791 | 787 | 793 | 792 | 793 | 794 | 787 | 787 | C+T, ring br. |  |
| 870 | 870 | 870 | 870 | - | - | - | - | G, in plane deformation ^4^ (shifted) |  |
| 958 | - | 955 | 955 | 958 | 958 | - | - | Ribose + phosphate;  A, NH_2_ rocking + N9C6 stretching ^5^ |  |
| 1030 | 1027 | 1030 | 1030 | 1030 | 1030 | - | - | Ribose + C |  |
| 1088 | 1089 | 1092 | 1095 | 1090 | 1090 | - | - | PO_2_^-^ symm. str. |  |
| - | - | - | - | - | - | 1128 | 1128 | A, C-N str. + N-ring ^5^ | Amino group |
| - | - | 1160 | 1160 | 1166 | 1166 | - | - | G, C-N str. ^5^ |  |
| 1195 | 1195 | 1195 | 1195 | - | - | 1183 | 1183 | G, C8-H bend. | Single bond stretching region |
| - | 1253 | - | 1253 | - | - | 1254 | 1254 | A, C-N str. ^5^ |  |
| 1328 | 1324 | 1328 | 1330 | 1328 | 1330 | 1322 | 1322 | A+G, ring modes |  |
| - | - | - | - | - | - | 1360 | 1360 | G, ring mode ^6^ |  |
| - | - | - | - | 1398 | 1398 | 1405 | 1405 | A, C-N str. ^5^ |  |
| 1420 | 1420 | 1408 | 1408 | 1414 | 1414 | 1416 | 1416 | backbone |  |
| - | - | - | - | - | - | 1436 | 1436 | backbone, CH_2_ deformation ^7^ |  |
| 1458 | 1451 | 1460 | 1458 | 1464 | 1460 | - | - | Ring modes, mainly C+T |  |
| 1486 | 1485 | 1492 | 1492 | - | 1487 | 1485 | 1485 | G+A ring modes, mainly G |  |
| 1508 | 1508 | - | - | - | - | 1507 | 1507 | G+A ring modes, mainly G |  |
| - | - | - | - | - | - | 1536 | 1536 | G, C-N str. ^5^ |  |
| 1578 | 1578 | 1577 | 1578 | 1573 | 1577 | 1576 | 1576 | G+A ring modes | Double bond stretching region |
| - | - | - | - | - | - | 1595 | 1595 | A, NH_2_ deformation ^8^ |  |
| 1636 | 1647 | 1634 | 1634 | 1638 | 1638 | 1647 | 1647 | T+C, v(C=O), mainly T | Carbonyl stretching |
| 1700 | - | - | - | - | - | - | - | T, v(C=O) ^2^ |  |

**Table S2.** Band positions for each nanoparticle system and for both uApt and hApt, together with the corresponding assignments from the literature, where A: Adenine, C: Cytosine, G: Guanine, and T: Thymine. Most of the bands are assigned by reference ^2^ where not otherwise specified.

**Figure S3.** Extinction spectra of AgSp, AgCitSp, and AgCitCl colloids after overnight incubation with uApt at different concentrations under the same conditions employed for SERS measurements.

**Figure S4.** Normalized SERS spectra acquired at 514 nm for mixtures of AgSp with increasing concentrations of the unfolded aptamer (uApt). Spectra are vertically offset for clarity. Dotted vertical lines mark selected Raman bands discussed in the text.

**Figure S5.** Normalized SERS spectra acquired at 514 nm for mixtures of AgSp with increasing concentrations of the hairpin aptamer (hApt). Spectra are vertically offset for clarity. Dotted vertical lines mark selected Raman bands discussed in the text.

**Figure S6.** Normalized SERS spectra acquired at 514 nm for mixtures of AgCitSp with increasing concentrations of the unfolded aptamer (uApt). Spectra are vertically offset for clarity. Dotted vertical lines mark selected Raman bands discussed in the text.

**Figure S7.** Normalized SERS spectra acquired at 514 nm for mixtures of AgCitSp with increasing concentrations of the hairpin aptamer (hApt). Spectra are vertically offset for clarity. Dotted vertical lines mark selected Raman bands discussed in the text.

**Figure S8.** Normalized SERS spectra acquired at 514 nm for mixtures of AgCitCl with increasing concentrations of the unfolded aptamer (uApt). Spectra are vertically offset for clarity. Dotted vertical lines mark selected Raman bands discussed in the text.

**Figure S9.** Normalized SERS spectra acquired at 514 nm for mixtures of AgCitCl with increasing concentrations of the hairpin aptamer (hApt). Spectra are vertically offset for clarity. Dotted vertical lines mark selected Raman bands discussed in the text.

**Figure S10.** Normalized SERS spectra acquired at 785 nm for mixtures of AgCitSp with increasing concentrations of the unfolded aptamer (uApt). Spectra are vertically offset for clarity. Dotted vertical lines mark selected Raman bands discussed in the text.

**Figure S11.** Normalized SERS spectra acquired at 785 nm for mixtures of AgCitSp with increasing concentrations of the hairpin aptamer (hApt). Spectra are vertically offset for clarity. Dotted vertical lines mark selected Raman bands discussed in the text.

**Figure S12.** Normalized SERS spectra acquired at 785 nm for mixtures of AgCitCl with increasing concentrations of the unfolded aptamer (uApt). Spectra are vertically offset for clarity. Dotted vertical lines mark selected Raman bands discussed in the text.

**Figure S13.** Normalized SERS spectra acquired at 785 nm for mixtures of AgCitCl with increasing concentrations of the hairpin aptamer (hApt). Spectra are vertically offset for clarity. Dotted vertical lines mark selected Raman bands discussed in the text.

**Figure S14.** Normalized SERS spectra of hApt before (untreated) and after TCEP treatment (TCEP-treated) on different colloidal substrates. (A) AgSp colloids, final aptamer concentration: 150 nM. Spectra were acquired using 633 nm excitation. (B) AgCitCl colloids, final aptamer concentration: 350 nM. Spectra were acquired using 785 nm excitation.

**Figure S15.** Aptamer concentration dependence (unfolded vs hairpin) of the ratiometric markers: A/G (ring breathing modes adenine vs guanine at 685 cm^-1^) and C/P (ring breathing mode of cytosine versus phosphate symmetric stretching), for the three colloidal platforms investigated (AgSp, AgCitSp, and AgCitCl). Solid lines are included as visual guides.

**Figure S16.** Aptamer concentration dependence (unfolded vs hairpin) of the ratiometric markers: A/C (ring breathing modes adenine vs cytosine), A/P (ring breathing mode of adenine versus phosphate symmetric stretching), and G/G (guanine stretching modes ~685 vs ~658 cm^-1^) for the three colloidal platforms investigated (AgSp, AgCitSp, and AgCitCl). Aptamer concentrations were normalized by the estimated total metallic surface area of each colloidal system, taking 70 nm diameter AgCitSp/AgCitCl nanoparticles as reference (relative surface area = 1). Solid lines are included as visual guides. Data points represent mean values obtained from the two independent experimental series (error bars indicate the standard deviation calculated from the corresponding values).

**Figure S17**. SERS spectra of uApt and hApt (25 nM) adsorbed on AgCitCl colloids aggregated with Al(NO_3_)_3_. The intense feature at 1048 cm^-1^ is ascribed to nitrate N-O stretching vibration.

**Figure S18.** Ratiometric analysis of selected SERS intensity ratios for uApt (25 nM) adsorbed on AgCitCl colloids under different preparation protocols. The bar plots compare the relative values obtained after overnight equilibration with the colloids prior to MgSO_4_ addition, 10 min pre-aggregation incubation, and prior overnight incubation of uApt with MgSO_4_ before colloid addition.

**REFERENCES**

(1)	Yguerabide, J.; Yguerabide, E. E. Light-Scattering Submicroscopic Particles as Highly Fluorescent Analogs and Their Use as Tracer Labels in Clinical and Biological Applications - I. Theory. *Anal. Biochem.* **1998**, *262* (2), 137–156.

(2)	Garcia-Rico, E.; Alvarez-Puebla, R. A.; Guerrini, L. Direct Surface-Enhanced Raman Scattering (SERS) Spectroscopy of Nucleic Acids: From Fundamental Studies to Real-Life Applications. *Chem. Soc. Rev.* **2018**, *47* (13). https://doi.org/10.1039/c7cs00809k.

(3)	Gisbert-Quilis, P.; Masetti, M.; Morla-Folch, J.; Fitzgerald, J. M.; Pazos-Perez, N.; Garcia-Rico, E.; Giannini, V.; Alvarez-Puebla, R. A.; Guerrini, L. The Structure of Short and Genomic DNA at the Interparticle Junctions of Cationic Nanoparticles. *Adv. Mater. Interfaces* **2017**, *4* (17). https://doi.org/10.1002/admi.201700724.

(4)	Gao, F.; Lei, J.; Ju, H. Label-Free Surface-Enhanced Raman Spectroscopy for Sensitive DNA Detection by DNA-Mediated Silver Nanoparticle Growth. *Anal. Chem.* **2013**, *85* (24), 11788–11793.

(5)	Otto, C.; van den Tweel, T. J. J.; de Mul, F. F. M.; Greve, J. Surface‐enhanced Raman Spectroscopy of DNA Bases. *J. Raman Spectrosc.* **1986**, *17* (3), 289–298. https://doi.org/10.1002/JRS.1250170311.

(6)	Torres-Nuñez, A.; Faulds, K.; Graham, D.; Alvarez-Puebla, R. A.; Guerrini, L. Silver Colloids as Plasmonic Substrates for Direct Label-Free Surface-Enhanced Raman Scattering Analysis of DNA. *Analyst* **2016**, *141* (17). https://doi.org/10.1039/c6an00911e.

(7)	Papadopoulou, E.; Bell, S. E. J. Label-Free Detection of Single-Base Mismatches in DNA by Surface-Enhanced Raman Spectroscopy. *Angew. Chem. Int. Ed.* **2011**, *50* (39), 9058–9061. https://doi.org/10.1002/anie.201102776.

(8)	Jang, N. H. The Coordination Chemistry of DNA Nucleosides on Gold Nanoparticles as a Probe by SERS. *Bull. Korean Chem. Soc.* **2002**, *23* (12), 1790–1800. https://doi.org/10.5012/bkcs.2002.23.12.1790.
